# Oral Selective Estrogen Receptor Degraders (SERDs) as a Novel Breast Cancer Therapy: Present and Future from a Clinical Perspective

**DOI:** 10.3390/ijms22157812

**Published:** 2021-07-22

**Authors:** Cristina Hernando, Belén Ortega-Morillo, Marta Tapia, Santiago Moragón, María Teresa Martínez, Pilar Eroles, Iris Garrido-Cano, Anna Adam-Artigues, Ana Lluch, Begoña Bermejo, Juan Miguel Cejalvo

**Affiliations:** 1 Hospital Clínico de València, Instituto de Investigación INCLIVA, 46010 Valencia, Spain; ortegamorillob@gmail.com (B.O.-M.); martapis3@gmail.com (M.T.); smoragon0@gmail.com (S.M.); maitemartinez3@yahoo.es (M.T.M.); iris_gc_255@hotmail.com (I.G.-C.); anna.adam.artigues@gmail.com (A.A.-A.); lluch_ana@gva.es (A.L.); begobermejo@gmail.com (B.B.); 2 Centro de Investigación Biomédica en Red de Oncología, CIBERONC-ISCIII, 28029 Madrid, Spain; 3 Departamento de Fisiología, Universidad de València, 46010 Valencia, Spain

**Keywords:** SERD, breast cancer, endocrine therapy, hormone therapy, luminal breast cancer

## Abstract

Estrogen receptor-positive (ER+) is the most common subtype of breast cancer. Endocrine therapy is the fundamental treatment against this entity, by directly or indirectly modifying estrogen production. Recent advances in novel compounds, such as cyclin-dependent kinase 4/6 inhibitors (CDK4/6i), or phosphoinositide 3-kinase (PI3K) inhibitors have improved progression-free survival and overall survival in these patients. However, some patients still develop endocrine resistance after or during endocrine treatment. Different underlying mechanisms have been identified as responsible for endocrine treatment resistance, where *ESR1* gene mutations are one of the most studied, outstanding from others such as somatic alterations, microenvironment involvement and epigenetic changes. In this scenario, selective estrogen receptor degraders/downregulators (SERD) are one of the weapons currently in research and development against aromatase inhibitor- or tamoxifen-resistance. The first SERD to be developed and approved for ER+ breast cancer was fulvestrant, demonstrating also interesting activity in *ESR1* mutated patients in the second line treatment setting. Recent investigational advances have allowed the development of new oral bioavailable SERDs. This review describes the evolution and ongoing studies in SERDs and new molecules against ER, with the hope that these novel drugs may improve our patients’ future landscape.

## 1. Introduction

Breast cancer (BC) is a heterogeneous disease comprising different subtypes, which can be identified through molecular biomarkers that also act as predictive factors. Luminal BC is characterized by the expression of estrogen receptor-positive (ER+) and/or progesterone receptor-positive (PR+), HER2-positive BC is defined by overexpression of human epidermal growth factor 2 (HER2) oncogene and conversely, triple-negative BC is characterized by lack of expression of ER/PR and HER2.

Among these, luminal is the most common BC subtype. In the case of metastatic breast cancer, luminal subtype accounts for more than sixty-five percent of all cases. Recommended treatment is endocrine-based systemic therapy, since multiple publications and consensus recommendations conclude that chemotherapy would not be the best option for endocrine sensitive disease, except in situations such as visceral crisis [1].

Endocrine therapy (ET) comprises different strategies as suppression of estrogen production or directly targeting the estrogen receptor (ER). For example, aromatase inhibitors (AIs) (letrozole, anastrozole and exemestane) are potent inhibitors of the aromatase enzyme, which catalyzes the last step in estrogen biosynthesis. These agents decrease estrogen production by blocking androgen conversion to estrogens [2,3].

Direct targeting of ERα is achieved by selective estrogen receptor modulator (SERM) (e.g., tamoxifen) and selective estrogen receptor degrader (SERD) (e.g., fulvestrant). SERMs compete with estrogen for ER binding and show mixed agonist/antagonist capabilities in a tissue-specific fashion. Meanwhile, SERDs create an unstable protein complex that induces ER protein degradation via proteasome [4]. Fulvestrant is a first-generation SERD approved by the FDA in 2007 for treatment of metastatic luminal BC in postmenopausal patients following progression on prior ET with AI or tamoxifen [5,6].

Several mechanisms of ET resistance have been described. Loss of ER expression occurs in only 10% of endocrine-resistant BC [7]. By contrast, estrogen-independent ER reactivation is the main mechanism of resistance [8]. This can occur through altered interactions of ER with coactivators/corepressors, via crosstalk between ER and other oncogenic signaling pathways [9], or by acquired mutations in *ESR1*. This mutation exists rarely in primary tumors (~1%) but is relatively common in metastatic ER+ disease representing up to 20% of patients who received aromatase inhibitors [10,11,12].

The recent addition of cyclin-dependent kinase 4/6 inhibitors (CDK4/6i) to ET has emerged as the first-line treatment option. This combination has improved prognosis in patients with advanced luminal BC compared with ET alone [1].

New SERDs are currently under development capable of reducing ERα protein expression and blocking estrogen-dependent and independent ER signaling. SERDs are therefore considered a significant therapeutic approach to treat ER+ BC in both early stage and more advanced drug-resistant cases.

This review aims to facilitate the understanding of the mechanisms of action of SERDs, and to summarize the ongoing development of new oral SERDs from a practical clinical perspective.

## 2. Antiestrogen Therapy: Basic Concepts Regarding Old and New Agents

Within the armamentarium for hormonal receptor-positive (HR+) BC, we find many different agents and diverse types of ET: aromatase inhibitors (AIs), SERMs and SERDs. These types of treatment are pivotal in the management of HR+ BC, and have demonstrated to improve both survival and relapsing times [13].

**Figure 1:**
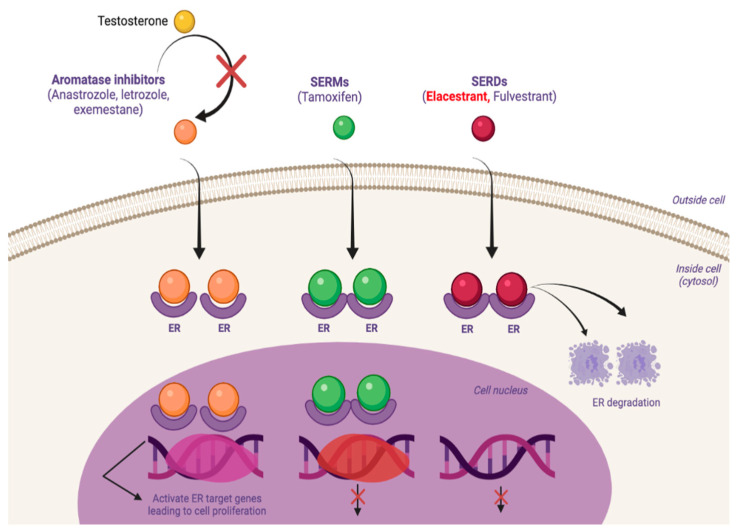
Mechanism of action of the different ET: aromatase inhibitors, SERMs and SERDs. ER and its activity modulated by AI, SERMs and SERDs: AI block estrogen production by inhibiting the aromatization of androgens to estrogens. SERMs (tamoxifen) competitively inhibit the binding of estrogen to ER. SERDs produce a reduction of SERD-bound ER ability to translocate to the nucleus, inhibiting transcription of ER-regulated genes. SERD-bound ER undergoes degradation as a consequence of impaired mobility.

AIs exert their action by blocking androgen to estrogen conversion, thus lowering the levels of circulating estradiol (E2) and, therefore, reducing the activation of ER. SERMs, such as tamoxifen, bind to intracellular ERs, competing against estrogen. These SERM-bound dimers interact with transcriptional factors, thus inhibiting the transcriptional activities in breast tissue. SERDs, such as fulvestrant, considered a pure ER antagonist, help to destabilize the ER by the SERD-bound ER that impedes to establish an open chromatin conformation that usually leads to transcription of ER regulated genes [14]. Furthermore, the SERD-ER bound causes impaired mobility that ends in the complex degradation, causing the downregulation and degradation of the receptor protein. (Figure 1)

### 2.1. ERα Structure

Two major isoforms of the estrogen receptor have been identified, ERα and ERβ: however, the role of ERβ in cancer remains unclear [15,16]. The two isoforms are encoded by two genes located on different chromosomes (*ESR1* on chromosome 6 and *ESR2* on chromosome 14), and regulate different specific genes [17,18]. Both isoforms are structurally organized in six different functional domains (A to F). The receptor contains two activation functions (AF) regions (AF-1: domains A/B and AF-2 domains E/F), responsible for the transcriptional activation of the receptor. C domain is the DNA-binding region, while D domain is a flexible hinge region containing the nuclear localization signal and links the C to E domain. Finally, E domain harbors the hormone-binding site [19].

ER is a transcription factor that regulates the expression of estrogen-responsive genes by binding to a specific DNA sequence found in their regulatory regions. This sequence is named the estrogen response element (ERE) [20,21]. Interaction of the estradiol-activated ERα dimer with EREs of genes constitutes the initial step in the ERE-dependent signaling pathway [22].

Furthermore, there are alternative noncanonical ER signaling pathways. For example, ER can interact with other transcription factors, such as AP-1 and Sp1, which will bind with non-ERE genes [19]. In addition, ER can also do its functions in the plasma membrane, where participates in the activation of different signaling cascade such as PI3K or MAPK [23,24]. Both canonical and noncanonical ER signaling are complementary and synergistic [25].

### 2.2. ER Alterations Driving Therapy Resistance: ESR1 Mutations

Several mechanisms regarding ER have been considered to drive resistance to anticancer drugs. Within these, alterations in *ESR1* are some of the most well-established and the main subject of interest to this date. *ESR1* mutations are characteristically more frequent in advanced disease, after endocrine therapy, rather than in primary BC [10,26].

While *ESR1* alterations, such as amplifications, can be identified in up to 30% of ER+ BC patients [27,28], it is still uncertain whether this alteration has clinical significance in terms of ET resistance: while some studies have found that *ESR1* amplifications were associated with improved disease-free survival [29,30] several others studies report an association between *ESR1* amplifications and tamoxifen resistance [31,32].

Similarly, clinical outcomes for ESR1 fusions require further investigation and efforts, since to this date conclusion cannot be drawn regarding their implications [14]. Fusions and rearrangements are estimated to have an incidence of 1%, mainly involving the first two noncoding exons of *ESR1* binding to various C-terminal sequences from the coiled-coil domain-containing 170 genes (CCDC170) (*ESR1*-e2 > CCDC170), consequently conferring endocrine resistance to tamoxifen [33].

Regarding mutations in *ESR1*, they are found in the ligand-binding domain, favoring constitutive ER activation independent from estrogen and resistance to AIs. [26]. However, *ESR1* mutated tumors can still present sensitivity to tamoxifen or fulvestrant [26,34]. Mutations in Y537S, Y537N and D538G are the most frequently identified.

A retrospective analysis of the SoFEA phase III trial showed that median PFS in fulvestrant-containing regimens was significantly better than those treated with exemestane (HR = 0.52; 95% CI 0.30–0.92; *p* = 0.02) for metastatic BC (MBC) and *ESR1* mutations [10]. This data may suggest that fulvestrant could be a potentially more adequate ET for *ESR1* mutated patients. Conversely, *ESR1* Y735S mutations may reflect higher resistance to fulvestrant [35,36].

More recently studies suggest a potential role of circulating *ESR1* mutations as a biomarker since these were linked to a higher risk of earlier progression in MBC patients during treatment with AIs [37].

### 2.3. Fulvestrant as First SERD

Fulvestrant is a pure antagonist of the ER which inhibits ER signaling by two mechanisms. It has demonstrated a higher affinity for ER than tamoxifen [38,39]. Binding to ER prevents ER dimerization and inhibits translocation of the receptor to the nucleus [40,41]. Moreover, the ER-fulvestrant complex is unstable allowing the degradation of the ER protein by the ubiquitin-proteasome system [42,43,44,45]. In 2002, fulvestrant was approved for MBC ER+ patients that had progressed on prior ET in the form of an intramuscular injection of 250 mg. However, the 500 mg dose demonstrated increased bioavailability and efficacy, and is currently the recommended approved dosage [5,6]. Limitations regarding the bioavailability of fulvestrant triggered research in new oral SERDs [46].

## 3. New ER-α Targeting Agents in Development

While ET has significantly reduced recurrence and mortality of BC patients, de novo and acquired resistance to this treatment remains a major challenge. Several new SERMs and SERDs are currently under clinical development. These drugs may overcome some of the limitations associated with current ET. Here, we review recent advances in potential strategies to overcome resistance to this therapy [47].

### 3.1. SERMs

SERMs are antiestrogenic agents that were developed to compete with estrogen and modulate ER activity by changing the binding coregulators and inhibiting ER-dependent transcriptional factors activity. SERMs can be classified based on their chemical structure as triphenylethylenes (tamoxifen and “tamoxifen-like”), benzothiophenes (raloxifene, arzoxifene), phenylindoles (bazedoxifene, pipindoxifene) and tetrahydronaphthalenes (lasofoxifene) [48].

Development of second- and third-generation SERMs expanded the structural diversity of ER binding molecules and achieved an antagonist effect in the breast tissue and bone protection activity, without uterotrophic activity. Unfortunately, all exhibited cross-resistance to tamoxifen and failed to demonstrate superiority over tamoxifen in clinical studies [49,50].

- LASOFOXIFENE

Lasofoxifene is a second-generation SERM which binds to ERα with similar potency and in a similar fashion as E2. This dimer causes a conformational change that prevents recruitment of co-activators [51].

Lasofoxifene has been extensively studied, especially in the setting of osteoporosis and gynecological health. In postmenopausal women with osteoporosis, the drug was associated with reduced risk of bone fractures, ER+ breast cancer, coronary heart disease, and stroke but increased risk of venous thromboembolic events [52,53].

Lasofoxifene has demonstrated preclinical antitumor activity in *ESR1*-mutant models, and indeed is currently being investigated in different clinical trials. The phase II ELAINE trial (NCT03781063) explores lasofoxifene versus fulvestrant in female patients with advanced luminal BC who have *ESR1* mutations. Moreover, the phase II ELAINE-2 trial (NCT04432454) is currently evaluating the combination of lasofoxifene plus abemaciclib in the same setting (Table 1).

- BAZEDOXIFENE

Bazedoxifene is a third-generation SERM. However, in some contexts, bazedoxifene displays a “SERD-like” profile, and is therefore it is often referred to as a mixed SERM/SERD hybrid [54,55]. Bazedoxifene has shown activity in tamoxifen-resistant xenografts and while this might be attributed to its SERD-like activity, it has been shown that the ER degradation is not a requirement for bazedoxifene’s antiestrogenicity [56].

Bazedoxifene is approved in Europe as monotherapy for prevention and treatment of osteoporosis. Its activity in BC has been studied both preclinically and clinically. In fact, bazedoxifene is being investigated in combination with palbociclib in advanced ER+ BC, based on preclinical data suggesting that these two drugs work synergistically in tumors with resistance to endocrine therapies, as well as in tumors that express *ESR1* mutations (NCT02448771) (Table 1).

### 3.2. Nonsteroidal Agents with Acrylic Acid Side Chain as Serds

**Figure 2:**
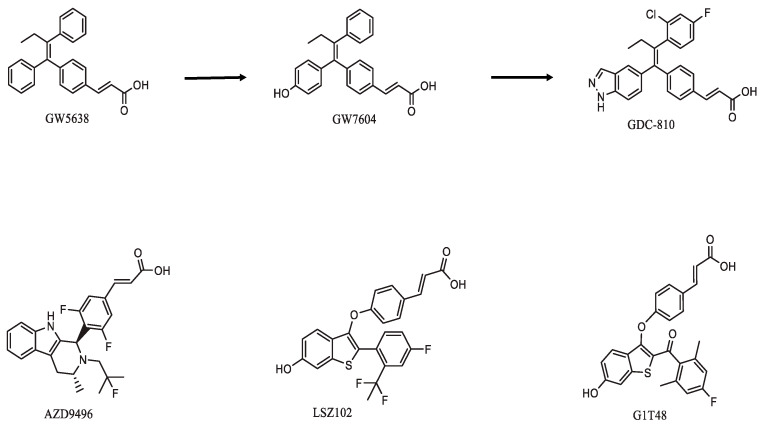
Structures of orally available SERDs with an acrylic acid functional group.

Optimization of ER degradation has been used as a strategy to identify lead compounds that possess a fulvestrant-like mode of action. These SERDS are nonsteroidal small molecules characterized by an ER binding motif and a side chain featuring either an acrylic acid or an amino base terminal that confer antiestrogenic and ER degrading activities [57] (Figure 2).

GW5638, a prodrug to GW7604, is a tamoxifen analog and was the first developed SERD that incorporated an acrylic acid side chain, as opposed to the tertiary amine group seen in other SERMs, including tamoxifen [58]. Unfortunately, GW5638 development did not progress forward, and there are currently attempts to discover oral SERDs of different structures from the molecular basis of GW5638, such as GDC0810, LSZ-102 and AZD9496, which have been tested in different clinical trials.

#### 3.2.1. GDC0810 (Brilanestrant)

Brilanestrant is one of the second-generation of nonsteroidal SERDs. It is a potent ERα-binder, a full transcriptional antagonist with no agonism and displays good potency and efficacy in ERα degradation [59,60]. However, when brilanestrant was compared with fulvestrant in a phase II clinical trial (NCT02569801), it failed to show comparable or superior efficacy.

#### 3.2.2. AZD9496

AZD9496 is an oral nonsteroidal small molecule and in preclinical data showed to be a potent and selective antagonist as well as a degrader of ERα. This drug also demonstrated antitumor activity in *ESR1*-mutant models [61].

AZD9496 was evaluated in a first-in-human phase I trial in ER+ BC patients. Forty-five patients with any menopausal status were treated with increasing doses of AZD9496 (from 20 mg once daily to 600 mg twice daily). AZD9496 was well tolerated with an acceptable safety profile, showing evidence of prolonged disease stabilization in heavily pretreated patients with ER+/HER2− advanced BC. Premenopausal patients were also treated with LHRH agonist. Patients received an average of three lines of prior ET in any setting: more than half of the patients received prior fulvestrant (55.6%), 40% received an mTOR inhibitor and only 7% were previously treated with CDK4/6i. The most common adverse events of any grade were diarrhea (35.6%), fatigue (31.1%), and nausea (22.2%) [62].

A preoperative window of opportunity study (NCT03236974) aimed to assess the biological effects of AZD-9496 (250 mg twice daily from day 1 for 5–14 days) versus fulvestrant (500 mg on day 1) in postmenopausal women with early ER+ BC. This was the first presurgical study to demonstrate that this SERD reduced Ki67, ER and PR expression. Although an advantage over fulvestrant was observed in preclinical data, AZD-9496 was not superior to fulvestrant at the tested dose [63].

#### 3.2.3. LSZ102

LSZ102 is another novel oral SERD discovered in 2018 as an acrylic acid SERD based on the benzothiophene scaffold [64]. It showed high potency and efficacy in its degradation profile as well as good pharmacokinetic performance. LSZ102 induced significant tumor regression in preclinical models [57].

LSZ102 is currently being evaluated in a phase I/Ib study of LSZ102 single agent and LSZ102 in combination with either ribociclib or alpelisib in patients with advanced ER+ BC who have progressed after ET (NCT02734615) (Table 1).

Across the three arms considered in this trial, 57.2% of patients received prior fulvestrant, 55.3% received prior CDK4/6i, and 67.96% received prior chemotherapy. In arm A, single-agent LSZ102 led to an objective response rate (ORR) of 1.3%, a clinical benefit rate (CBR) of 9.1%, and a median PFS of 1.8 months (95% CI, 1.7–2). In arm B, the combination of LSZ102 plus ribociclib elicited a 15.8% ORR and a CBR of 35.5%; the median PFS was 6.2 months (95% CI, 4.4–6.4). When LSZ102 was combined with alpelisib, the ORR was 5.4%, the CBR was 18.9%, and the median PFS was 3.5 months (95% CI, 1.8–5.5). Regarding safety, results showed that the most frequent adverse events across all three arms were gastrointestinal toxicity (nausea, vomiting, and diarrhea). In arm B, grade 3 neutropenia was described in 13.2% of the patients and was likely driven by ribociclib. In arm C, grade 3 hyperglycemias (10%), and skin rashes (15.4%) were reported and were likely driven by alpelisib. In all three arms, the genomic landscape was dominated by *ESR1*, *PIK3CA*, and *TP53* mutations in baseline circulating tumor DNA (ctDNA). However, these mutations did not correlate with response and were not enriched upon progression in patients with matched baseline and end-of-treatment samples [65] (Table 2).

#### 3.2.4. G1T48 (Rintodestrant)

G1T48 is another example of an orally bioavailable nonsteroidal agent. This molecule is a potent and efficacious inhibitor of estrogen-mediated transcription and proliferation in ER+ BC cells, similar to fulvestrant. In addition, G1T48 can effectively suppress ER activity in multiple ET resistance models including those harboring *ESR1* mutations and growth factor activation. These data showed that G1T48 has the potential to be an efficacious oral antineoplastic agent in ER+ BC alone or in combination with CDK4/6i [66,67].

An ongoing phase I trial including 96 patients in both dose-escalation and dose-expansion cohorts is evaluating the tolerability and preliminary efficacy of G1T48 in endocrine-resistant MBC patients, in combination with palbociclib or monotherapy (NCT03455270) (Table 1).

### 3.3. Nonsteroidal Analogs with a Basic Amino Side Chain as SERDs

**Figure 3:**
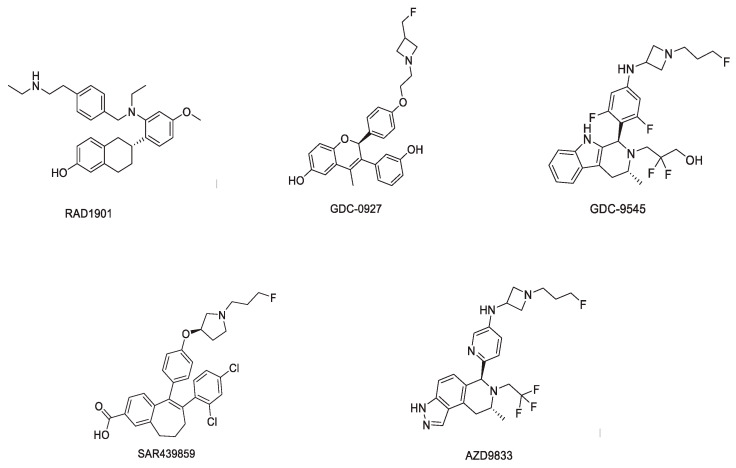
Structures of oral SERDs with a basic side chain.

Compared to acrylic-acid-containing oral SERDs that do not degrade ER equally in different ER+ cell lines, basic SERDs were optimized to deliver maximal ERα degradation across multiple ER+ cell lines, a feature possessed by fulvestrant. This new SERDs has oral and brain bioavailability, while maintaining high-affinity binding to ERα and both potency and efficacy comparable to fulvestrant in ET-resistant or *ESR1*-mutated cell lines [19,68]. (Figure 3)

#### 3.3.1. RAD1901 (Elacestrant)

Elacestrant (RAD1901) is a basic amino side chain SERD, first reported in 2015, which selectively binds to ER and induces its degradation leading to the inhibition of downstream signaling [69].

In preclinical models, elacestrant displayed a dose-dependent tumor reduction. Moreover, the effect was observed in multiple ER+ PDX models including those originating from patients who previously received multiple lines of ET. Interestingly, elacestrant also showed activity in *ESR1* mutant and in CDK4/6i-resistant models [70,71,72].

These preclinical data have formed the basis for several clinical studies. Recently, Bardia et al. published the phase I study results (NCT02338349) of elacestrant in postmenopausal women with heavily pretreated ER+/HER2- MBC, including those with *ESR1* mutations. The recommended phase II dose (RP2D) was 400 mg once daily. The median age was 63 years, and received an average of three prior lines of therapy, including CDK4/6i (52%), SERD (52%). *ESR1* mutations were detected in 50% of patients at (ctDNA. No dose-limiting toxicity (DLT) occurred and the most common adverse events were grade 1–2 severity: nausea (33.3%), increased blood triglycerides and decreased blood phosphorus (25.0% each). The ORR was 19.4%; 15.0% in patients with prior SERD, 16.7% in patients with prior CDK4/6i, and 33.3% in patients with *ESR1* mutation. The clinical benefit rate (24-week) was evaluable in 47 patients receiving RP2D, being 42.6% overall; 56.5% with *ESR1* mutation, and 30.4% with prior CDK4/6i. The clinical benefit was associated with a decline in *ESR1* mutant allele fraction. This study concluded that elacestrant 400 mg orally once daily has an acceptable safety profile and demonstrated single-agent activity with confirmed partial responses in heavily pretreated patients with ER+ MBC, in patients with *ESR1* mutation as well as those with prior CDK4/6i and prior SERD [73] (Table 2).

Elacestrant efficacy is currently being evaluated in a phase III clinical trial (NCT03778931). This is the first oral SERD tested in a phase III study. The objective is compare the efficacy and safety of elacestrant vs. endocrine monotherapy treatment (fulvestrant or AI) in postmenopausal ER+ HER2- MBC patients. Patients may be included with one or two prior lines of ET including a combination with CDK 4/6 inhibitor and no more than one line of chemotherapy. The role of *ESR1* mutation will be evaluated [74] (Table 1).

#### 3.3.2. GDC-0927

Further optimization of ERα degradation was achieved with GDC-0927 [75,76]. The safety of this new compound was assessed in a first-in-human phase I study performed in the United States and Spain including 42 postmenopausal patients (12 in the dose-escalation and 30 in the dose expansion cohort) who had received maximum two prior lines of ET (NCT02316509). Patients were treated with once-daily doses of GDC-0927 constituting between 600 and 1400 mg. Almost half of them were exposed to fulvestrant before study entry (43%) and 45% to CDK 4/6 inhibitors. *ESR1* mutation was detected at baseline plasma samples in 20 patients (48%). Tolerance was excellent. In fact, maximum tolerated dose was not reached and the phase 2 recommended dose was 1400 mg. The most frequent G1–G2 adverse events were nausea (19%), vomiting (10%), diarrhea (14%) and hot flushes (14%). On treatment, biopsies showed reduction of ER/PR levels and proliferation including in *ESR1* mutant tumors. In patients treated with 1400 mg, 13% had an unconfirmed response and CBR was 36%. Interestingly, allele frequency of *ESR1* and *PIK3CA* mutations in ctDNA declined in the majority of baseline-positive patients. Furthermore, 7 out of 10 patients showing clinical benefit had complete elimination of ctDNA on D1C3. ER pathway suppression was observed at both the transcript and protein level [77,78].

However, the clinical development of GDC-0927 has been halted due to suboptimal drug-like properties (NCT02316509).

#### 3.3.3. GDC-9545 (Giredestrant)

GDC-9545 was developed to address the poor clinical performance of the acrylic acid SERD GDC-0810, and first-generation basic SERD GDC-0927. It is highly potent competing against E2 for binding, and in driving an antagonist conformation within the ER ligand binding domain, induces ER turnover, and suppresses ER transcriptional activity, resulting in robust antiproliferative effect. In addition to its direct antagonist properties, the mechanism of action of GDC-9545 includes reducing levels of ER protein through proteasome-mediated degradation. GDC-9545 data demonstrated robust nonclinical activity in ER+ BC models of both *ESR1*−wild type and *ESR1*-mutated disease [79,80].

GDC-9545 is currently being evaluated in multiple ongoing clinical trials:

GO39932 is a Phase Ib/II, open-label, multicenter, randomized umbrella study in BC patients. The dose-escalation part of the trial recommended 100 mg once daily to be explored. Results from the dose-expansion cohort included [81]: 88 patients (cohort A: giredestrant monotherapy, n = 40; cohort B: giredestrant plus palbociclib, n = 48). Patients from cohort A exhibited a median ORR of 13% (95% CI, 4–30%), and a median PFS of 7.8 months (95% CI, 5.3–11.4). GDC-9545 was well tolerated, with more frequently grade 1 or 2 treatment-emergent adverse events (TRAEs), and no discontinuations. Patients from cohort B showed a median ORR of 33% (95% CI, 20–49%), and a median PFS of 9.3 months (95% CI, 8.9-not evaluable), within the 43 patients able to be evaluated. Most AEs within the combination cohort were also grade 1 or 2 severities, with 1 grade 3 AE, consisting of a chemotherapy prolongation in a patient who had a preexisting coronary artery condition. (Table 1 and Table 2).

AcelERA (WO42312) is a phase II, randomized, open-label, multicenter study evaluating the efficacy and safety of giredestrant compared to physician’s choice of endocrine monotherapy in participants with ER+/HER2-, locally advanced or MBC who have received one or two prior lines of systemic therapy in the locally advanced or metastatic setting (NCT04576455). CoopERA study is a window-of-opportunity phase II trial evaluating efficacy, safety and pharmacokinetics of giredestrant versus anastrozole and giredestrant plus palbociclib compared with anastrozole plus palbociclib in postmenopausal women with untreated, early ER+/HER2- BC (NCT04436744) (Table 1).

PersevERA (BO41843) is a phase III, randomized, double-blind, placebo-controlled, multicenter study that will evaluate the efficacy and safety of giredestrant combined with palbociclib compared with letrozole combined with palbociclib in patients with ER+/HER2- locally advanced (recurrent or progressed) or MBC (NCT04546009) (Table 1).

Lastly, GO40987 is a Phase I study evaluating the pharmacodynamics, pharmacokinetics, safety, and biologic activity of giredestrant in participants with operable stage I-III untreated ER+ HER2- BC (NCT03916744) (Table 1).

#### 3.3.4. SAR439859 (Amcenestrant)

Amcenestrant (SAR439859) is a potent, orally bioavailable, and selective ERα inhibitor that belongs to the SERD class of compounds. Amcenestrant antagonizes the binding of E2 to ER but also promotes the transition of ERα to an inactive conformation that leads to up to 98% receptor in in vitro assays. These dual properties lead to a deeper inhibition of ERα pathways and a more effective antiproliferative activity in ERα-dependent BC cell lines driven by mutant or wild-type ERα compared to other ERα inhibitors [82,83].

The use of amcenestrant as monotherapy has shown encouraging signals in the ongoing phase 1/2 AMEERA-1 trial (NCT03284957). In interim results reported at the 2020 San Antonio Breast Cancer Symposium (SABCS), amcenestrant monotherapy elicited antitumor activity in heavily pretreated, postmenopausal women with ER+ advanced or MBC [84] (Table 1).

Results showed that the ORR was 8.5% with amcenestrant achieving a CBR of 33.9% among pooled results from 59 patients who received amcenestrant at 150 mg or more daily. In a cohort of 33 patients who had received three or fewer prior lines of therapy in the metastatic setting, the ORR was 15.2% and the CBR was 42.4%. Moreover, in a subgroup of 14 patients who did not receive prior CDK4/6i, mTOR inhibitors, or fulvestrant, the ORR was 21.4% and the CBR was 64.3%. In part A of the trial, which was the dose-escalation phase, investigators evaluated amcenestrant at once daily doses ranging from 20 mg to 600 mg. In part B, which was the dose-expansion phase, the recommended dose for amcenestrant as monotherapy was determined to be 400 mg once-daily. Amcenestrant was found to have a favorable safety profile with 62.9% of patients experiencing TRAEs, none of which were grade 3 or higher. The most common (≥5%) TRAEs in the pooled population of patients who were treated with amcenestrant at the 150-mg or higher daily dose included hot flushes (16.1%), constipation (9.7%), arthralgia (9.7%), decreased appetite (8.1%), vomiting (8.1%), diarrhea (8.1%), nausea (8.1%), and fatigue (6.5%) (Table 2).

Amcenestrant monotherapy is also being evaluated in comparison to physician’s choice of therapy in the open-label, phase 2 trial AMEERA-3 trial (NCT04059484), which will enroll 372 patients. The control treatment involves choosing monotherapy from a list of agents with different mechanisms of action: anastrozole, letrozole, or exemestane, tamoxifen, or fulvestrant (Table 1).

AMEERA-5 study (NCT04478266), is testing amcenestrant in combination with palbociclib, a CDK4/6i versus letrozole plus palbociclib as a first-line therapy for patients with ER+, HER2- locoregional or MBC (Table 1).

AMEERA-4 is a phase 2 “window of opportunity” study that tests two dose levels of amcenestrant versus letrozole given for 14 days to patients with ER+, HER2- localized BC who are candidates for breast-conserving therapy or upfront mastectomy. The study will measure the impact of the short course of endocrine therapy on Ki67. Ki67 expression has been correlated with poor cancer-specific survival at a cutoff point greater than 14% of tumor nuclei [85] (Table 1).

#### 3.3.5. AZD9833 (Camizestrant)

AZD9833 is a potent, selective, nonsteroidal, pure ERα antagonist and SERD that can be administered orally. Compared to AZD9496, AZD9833 is a better ERα degrader [86].

Preclinically, this drug has demonstrated equivalent maximal ERα degradation to fulvestrant in a panel of ER+ BC cell lines and complete antagonism of estradiol-induced gene expression changes in vitro. Furthermore, AZD9833 did not cause any agonism of ER activity in the absence of E2 in ER+ BC, nor uterine endothelial models in vitro and in vivo. AZD9833 caused significant antitumor effects in several PDX models of ER+ BC, including those bearing clinically relevant mutations in *ESR1* [86].

SERENA-1 study (NCT03616587) is currently assessing AZD9833 as monotherapy and in combination with other anti-cancer therapies in pretreated luminal MBC. Results reported in SABCS 2020 [87] showed that ORR and CBR for monotherapy were 10.0% and 35.3%, respectively, and median PFS was 5.4 months. In CDK4/6i naïve patients treated with combination therapy, the ORR and CBR were 14.3% and 71.4%, respectively. The majority of treatment-related adverse effects were of grade 1 or 2 severity and most commonly included anemia, fatigue, lymphopenia, nausea, neutropenia, thrombocytopenia, and reduced white blood cell count. No patients discontinued treatment due to adverse events (Table 1 and Table 2).

Based on this very encouraging preclinical and early clinical data with AZD9833 a phase 2 study comparing the efficacy and safety of AZD9833 versus fulvestrant (SERENA-2; NCT04214288), a presurgical “window of opportunity” study (SERENA-3; NCT04588298) and a phase 3 study comparing the effects of AZD9833 and palbociclib versus anastrozole and palbociclib as an initial treatment for women with ER+ HER2– advanced BC (SERENA-4; NCT04711252) have commenced and form part of a comprehensive development program for this agent. (Table 1)

#### 3.3.6. Others

LY3484356 is a potent degrader and selective pure antagonist of wild type and mutant ERα [88]. It is currently being studied in the first-in-human, multicenter phase 1a/1b EMBER trial in patients with ER+ locally advanced or MBC (NCT04188548), including a dose-escalation phase as monotherapy (n = 100), followed by dose expansion (n = 360) as monotherapy and in combination with other therapies [88]. Preliminary results were reported from 28 patients. The most frequent toxicities were nausea (32%), fatigue (25%), and diarrhea (18%). At the first data cutoff, among 16 evaluable patients, 11 had stable disease (SD) 10 patients ongoing, and 5 had progressive disease (PD) [89] (Table 1 and Table 2).

A phase 1 EMBER-2 trial in preoperative, postmenopausal women with stage I-III, ER+/HER2- BC is also ongoing (NCT04647487) (Table 1).

ZN-c5 is a small molecule with potent antagonism and degradative properties against the ER both in vitro and in vivo, and showed a high oral bioavailability across several preclinical species as compared to other SERDs. To test if the high oral bioavailability can be translated to potent efficacy in vivo, the antitumor activity of ZN-c5 was evaluated in MCF-7 orthotopic tumor xenograft model. Oral ZN-c5 treatment at 5 mg/kg and 10 mg/kg resulted in 89% and 102% tumor growth inhibition, respectively. The combination of ZN-c5 with cell cycle inhibitors such as CDK4/6i or PI3K inhibitors resulted in enhanced antitumor activity. In addition to MCF-7 model, the activity of ZN-c5 in ER-mutant models including WHIM20, a Y537S *ESR1* patient-derived xenograft model, was evaluated, resulting in an induced 64% tumor growth inhibition at 40 mg/kg dosing while fulvestrant at 200 mg/kg (eightfold higher than that achieved in the clinic) resulted in 13% tumor growth inhibition. These data indicate that ZN-c5 has improved antitumor activity over fulvestrant in human tumor xenograft models. (1) Zn-c5 is currently in clinical trials as a single agent and in combination studies. NCT03560531 is a Phase 1/2, open-label, multicenter, dose-escalation and expansion study to evaluate the safety, tolerability, pharmacokinetics, and preliminary efficacy of ZN-c5 administered orally in subjects with advanced ER+ HER2- BC [90]. (Table 1).

D-0502 is another oral SERD discovered and currently being evaluated in a phase I clinical trial, an ongoing open-label study of D-0502 single agent and in combination with palbociclib, aiming to assess the safety and tolerability and also to evaluate preliminary antitumor activity in women with ER+, HER2- advanced or MBC (NCT03471663) (Table 1).

## 4. Novel Strategies

### 4.1. Proteolysis Targeting Chimera (PROTAC) as a New Class of Serd

**Figure 4:**
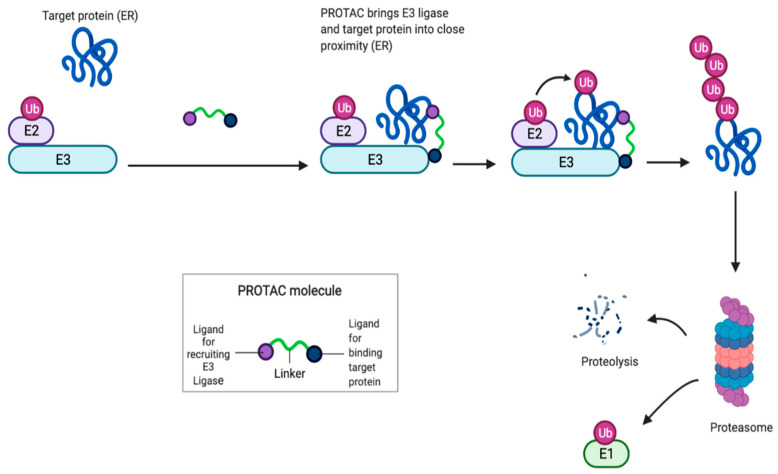
PROTACs: proteolysis targeting chimeras are heterobifunctional molecules made up of a ligand for ER (target protein) and another ligand, serving as the E3 ubiquitin ligase complex substrate. Once PROTACs bind to ER, recruit the E3 ubiquitin ligase complex, leading to a polyubiquitilation of ER ending on a proteasomal degradation.

PROTACs are heterobifunctional molecules made up of a ligand for ER (target protein) and another ligand, serving as the E3 ubiquitin ligase complex substrate. Once PROTACs bind to ER, recruit the E3 ubiquitin ligase complex, leading to a polyubiquitilation of ER ending on a proteasomal degradation [91]. PROTACs produce a rapid and complete elimination of intracellular receptor and inhibition of ER signaling [92,93]. PROTACs action is pure antagonism of ER realized by elimination of the receptor, rather than conformational changes of ER to block transcriptional activation. Only a transient binding event is required for degradation, and the PROTAC molecules can cycle through multiple rounds of activity, removing substoichiometric quantities of proteins. (Figure 4)

The rapid progress in ER PROTACs development in preclinical studies lead to a first-in-class, orally bioavailable ER degrading agent, ARV-471, which entered clinical trials in 2019 (NCT04072952) (Table 1).

ARV-471 is a PROTAC in which E2 is linked to a small-molecule ubiquitin E3 ligase–binding moiety, facilitating the interaction between the ER and an E3 ligase complex that will tag the ER for degradation by the ubiquitin-proteasome system [94].

In a December 2020 press release, Arvinas detailed interim findings from a phase 1 clinical trial of ARV-471 (NCT04072952). As of November 2020, 21 patients had completed at least one treatment cycle. Patients were heavily pretreated with a median of five prior lines of therapy. Results included one confirmed partial response (PR), two unconfirmed PR, and a CBR of 42%. The most common TRAEs were grade 1 or 2 nausea, arthralgia, fatigue, and decreased appetite. A cohort expansion in which ARV-471 is administered in combination with palbociclib is ongoing [95] (Table 1).

### 4.2. Selective Estrogen Receptor Covalent Antagonists (Serca)

A novel class of ERα inhibitors called selective ERα covalent antagonists (SERCAs) was introduced by Puyang et al. in 2018. Looking for a compound that could be either covalent or noncovalent ligands of the mutated ERα, Puyang et al., identified H3B-5942, the first-in-class SERCA developed by H3 Biomedicines. It covalently binds the ERα C530 residue both in ER-wild type and mutant settings (e.g., Y537S, D538G) and forcing ERα to fold towards a unique antagonist conformation, suppressing ERα-dependent transcription in BC cells in a different way from that of SERMs and SERDs [96].

H3B-5942 was tested and well-tolerated in experimental mice in vivo, against different BC tumor models including ERα-wild type and ERα-mutated, demonstrating strong antiproliferative activity and it showed superiority to fulvestrant in ER+ BC models. The antitumor effects of H3B-5942 can be enhanced when administered in combination with CDK4/6i or mTOR inhibitors in both ER-wild type and ER-mutated cell lines and/or tumor models [96].

A phase 1/2 clinical trial (NCT03250676) of H3B-6545 in pre- or postmenopausal women with previously treated advanced BC was developed. H3B-6545 demonstrated a manageable safety profile and single-agent antitumor activity in heavily pretreated ER+, HER2- MBC patients including those with a constitutively active clonal *ESR1* Y537S mutation (Table 1).

A total of 130 patients were enrolled (47 in the phase I part and 83 in the phase II part of the trial) and 105 (58% ER1-mutated) were response-evaluable. The phase I evaluated once daily doses from 100 to 600 mg and the dose of 450 mg was selected as the RP2D. Median age was 62 years and in MBC, the median number of prior therapies was three. Prior CDK4/6i, fulvestrant, and chemotherapy were received by 87%, 71%, and 54% of the patients, respectively. Regarding toxicities, grade 2 or higher adverse events reported in ≥10% were anemia (20%), fatigue (16%), nausea (14%), diarrhea (11%) and AST increase (11%). In the response-evaluable group, 13 confirmed PR (12%). SD and CBR (≥23 weeks) were 45% and 33% respectively at 450 mg. Three PRs (25%) and four SDs were observed in 12 patients in whom clonal *ESR1* Y537S was present. Median PFS in all patients was 3.7 months and in *ESR1*-mutated patients (Y537S) was 7.3 months [97] (Table 2).

There is currently an ongoing open-label, multicenter, phase 1b study of H3B-6545 in combination with palbociclib in women with advanced or metastatic ER+ HER2- BC (NCT04288089). (Table 1)

## 5. Summary

ER is involved in the initiation of BC tumorigenesis and in the progression of disease after ET. Targeting, modulating, and degrading ER is the goal of new drugs development, including *ESR1* gene mutations identified after ET. Fulvestrant is the only approved SERD and can be used in first-line treatment or after AI or tamoxifen progression. Overcoming fulvestrant’s limitations, new SERDs are currently in early-phase clinical development and some of them in phase III clinical trials. New SERDs have demonstrated improved pharmacokinetic and bioavailability compared to fulvestrant in preclinical and early studies, with a potentially higher clinical benefit rate. In this line, PROTACs and SERCAs open new paths to degrade ER, and are still in early clinical studies.

All the currently available results need to be confirmed in phase III clinical trials with larger patient population, exploring the activity of ET plus CDK4/6i combination progression disease setting.

## Figures and Tables

**Table 1:** Ongoing Trials: Numerous nonsteroidal SERDs are now being studied in clinical trials. Here we summarize the orally available SERDs currently in clinical development.

AGENT	TREATMENT	DISEASE SETTING	PHASE	NAME (INDICATOR)
SERMs				
LASOFOXIFENE	vs. FULVESTRANT +ABEMACICLIB	Previously treated advanced/metastatic disease with * ESR1 * mutations Previously treated advanced/metastatic disease with * ESR1 * mutations	2 2	ELAINE: NCT03781063 ELAINE 2: NCT04432454
BAZEDOXIFENE	+PALBOCICLIB	Previously treated advanced/metastatic	1/2	NCT02448771
SERDs				
LSZ102	SINGLE AGENT/+ RIBOCICLIB/+ ALPELISIB	Previously treated advanced/metastatic	1/1b	NCT02734615
G1T48 (RINTODESTRANT)	SINGLE AGENT+/− PALBOCICLIB	Previously treated advanced/metastatic	1	NCT03455270
RAD1901 (ELACESTRANT)	vs. SOC (standard of care)	Previously treated advanced/metastatic	3	EMERALD: NCT03778931
GDC-9545 (GIREDESTRANT)	GDC-9545 vs. LETROZOLE + PALBOCICLIB GDC-9545 vs. ANASTROZOLE + PALBOCICLIB vs. physician’s choice of endocrine therapy +/− PALBOCICLIB and LHRH agonist Monotherapy	Advanced/metastatic Treatment-naïve early breast cancer (window-of-opportunity -> neoadjuvant) Previously treated advanced/metastatic Advanced/metastatic Treatment-naïve early breast cancer (window-of-opportunity)	3 2 2 1 1	persevERA: NCT04546009 coopERA: NCT04436744 acelERA: NCT04576455 NCT03332797 NCT03916744
SAR439859 (AMCENESTRANT)	SAR439859 vs. LETROZOLE + PALBOCICLIB vs. physician’s choice of endocrine therapy vs. LETROZOLE +/− PALBOCICLIB OR ALPELISIB	Advanced/metastatic Previously treated advanced/metastatic Newly diagnosed advanced/metastatic Advanced/metastatic	3 2 2 1/2	AMEERA-5: NCT04478266 AMEERA-3: NCT04059484 AMEERA-4: NCT04191382 AMEERA-1 NCT03284957
AZD9833 (CAMIZESTRANT)	AZD9833 vs. LETROZOLE + PALBOCICLIB MONOTHERAPY vs. FULVESTRANT +/− PALBOCICLIB, EVEROLIMUS OR ABEMACICLIB	Treatment-naïve advanced/metastatic Neoadjuvant treatment Previously treated advanced/metastatic Previously treated advanced/metastatic	3 2 2 1	SERENA-4: NCT04711252 SERENA-3: NCT04588298 SERENA-2: NCT04214288 SERENA-1: NCT03616587
LY3484356	+/− other anticancer therapies MONOTHERAPY	Advanced/metastatic Neoadjuvant treatment	1 1	EMBER: NCT04188548 EMBER 2: NCT04647487
Zn-c5	SINGLE AGENT +/− PALBOCICLIB	Previously treated advanced/metastatic	1/2	NCT03560531
D-0502	SINGLE AGENT +/− PALBOCICLIB	Previously treated advanced/metastatic	1	NCT03471663
NOVEL THERAPIES				
ARV-471 (PROTAC)	+/− PALBOCICLIB	Previously treated advanced/metastatic	1/2	NCT04072952
H3B-5942 (SERCA)	MONOTHERAPY + PALBOCICLIB	Previously treated advanced/metastatic Previously treated advanced/metastatic	1/2 1	NCT03250676 NCT04288089

Abbreviations: ORR: overall response rate; CBR: clinical benefit rate; PFS: progression-free survival; AEs: adverse events; AAT: aspartate aminotransferase; DLT: dose-limiting toxicities; SD: stable disease.

**Table 2:** Reported efficacy and toxicity: Efficacious ER target engagement and promising clinical activity was shown in early-phase clinical trials with a good toxicity profile but clinical efficacy needs to be confirmed in larger patient populations.

LSZ102	Phase I/Ib (NCT02734615) (65) Arm A: Monotherapy Arm B: Combination with Arm B: Combination with Alpelisib	Arm A (n: 78): ORR (1.3%), CBR (9.1%), PFS (1.8 m) Arm B (n: 76): ORR (15.8%), CBR (35.5%), PFS (6.2 m) Arm C (n: 39): ORR (5.4%), CBR (18.9%), PFS 3.5m	Arm A, B, C: Grade 3/4: Nausea (3.1%) and Diarrhea (6.7%) Arm B (Grade 3 AEs): Neutropenia (13.2%) and Increased AAT (3.9%) Arm C (Grade 3 AEs): Hyperglycemia (10%), Skin Rashes (15.4%)
RAD1901 (ELACESTRANT)	Phase I (NCT02338349) (73)	N: 50 (dose-escalation) ORR 19.4% N: 47 (dose expansion) CBR 42.6%	No DLTs Grade 1/2: nausea (33.3%), increased triglycerides (25%), decreased blood phosphorus (25%)
GDC-9545 (GIREDESTRANT)	Phase Ib/II (NCT03332797) (88) Dose expansion: Cohort A: monotherapy Cohort B: combination with palbociclib	N: 88 Cohort A, n: 39 ORR (13%), PFS (7.8 m) Cohort B, n:43 ORR (33%), PFS (9.3 m)	Cohort A: Grade 1/2 fatigue, arthralgia. Grade 3: Fatigue (1), diarrhea (1), transaminase increased (1) Cohort B: Grade 1/2: neutropenia, fatigue, bradycardia, diarrhea, constipation, dizziness, nauseas, anemia, asthenia, pruritus and visual impairment. Grade 3: neutropenia (50%)
SAR439859 (AMCENESTRANT)	Phase I: AMEERA-1 (NCT03284957). (84) Monotherapy dose-escalation (Part A)	Part A: n: 59 ORR 8.5%, CBR (33.5%)	Part A: hot flushes (16.1%), constipation (9.7%), arthralgia (9.7%), decreased appetite (8.1%), vomiting (8.1%), diarrhea (8.1%), nausea (8.1%), and fatigue (6.5%)
AZD9833 (CAMIZESTRANT)	Phase I: SERENA-1 (NCT03616587) (87) Part A and B: monotherapy Part C and D: Combination with palbociclib	Part A and B, n: 98 ORR (10%), CDR (35.3%), PFS (5.4m) Part B and C, n: 48 ORR (6.3%), CBR (50%)	5 dose-limiting toxicities (3 for monotherapy and 2 for combination therapy) Monotherapy (≥Grade 2 instances of AZD9833-related adverse events): fatigue (9%), bradicardia (3.1%), nausea (3%), visual disturbances (1.1%) Combination: grade 1–2: anemia, fatigue, lymphopenia, nausea, neutropenia, thrombocytopenia, and reduced white blood cell count
LY3484356	Phase I/Ib (89) EMBER (NCT04188548)	N: 28	Grade 1–2: nausea (32%), fatigue (25%), and diarrhea (18%)
H3B-5942 (SERCA)	Phase 1/2 (NCT03250676) (97)	N: 130 (phase I n: 47/phase II n: 83) PR (12%). SD (45%) and CBR (33%), PFS (3.7m)	Grade 2 or higher adverse: anemia (20%), fatigue (16%), nausea (14%), diarrhea (11%) and AST increase (11%).

## Abbreviations

AF	Activation function
AEs	Adverse events
ALT	Alanine aminotransferase
AIs	Aromatase inhibitors
AST	Aspartate aminotransferase
ctDNA	Circulating tumor DNA
CBR	Clinical benefit rate
CDK4/6i	Cyclin-dependent-kinase 4/6 inhibitors
DNA	Deoxynucleic acid
DLT	Dose limiting toxicity
ET	Endocrine therapy
E2	Estradiol
ER	Estrogen receptor
ERE	Estrogen responsive element
ER+	Estrogen receptor-positive
ERK	Extracellular signal regulated kinase
HR	Hormone receptor
HR+	Hormonal receptors-positive
HER2	Human epidermal growth factor receptor 2
LFTs	Liver function tests
LTED	Long-term estrogen-deprived
MTD	Maximum tolerated dose
MBC	Metastatic breast cancer
MAPK	Mitogen-activated protein kinase
ORR	Objective response rate
PR	Partial response
PDX	Patient-derived xenograft
PK	Pharmacokinetics
RP2D	Phase II dose
PI3K	Phosphoinositide 3-kinase
PR+	Progesterone receptor-positive
PFS	Progression-free survival
PD	Progressive disease
AKT	Protein kinase B
PROTAC	Proteolysis targeting chimera
SERCA	Selective estrogen receptor covalent antagonists
SERD	Selective estrogen receptor degraders
SERM	Selective estrogen receptor modulator
SD	Stable disease
SOC	Standard of care
TRAEs	Treatment-emergent adverse events
